# Definition of immune molecular subtypes with distinct immune microenvironment, recurrence, and PANoptosis features to aid clinical therapeutic decision-making

**DOI:** 10.3389/fgene.2022.1007108

**Published:** 2022-10-13

**Authors:** Sufeng Qiang, Fei Fu, Jianjun Wang, Chunyan Dong

**Affiliations:** ^1^ Department of Gynaecology and Obstetrics, Shanghai East Hospital, Nanjing Medical University, Shanghai, China; ^2^ Department of Gynaecology and Obstetrics, Shanghai East Hospital, Tongji University School of Medicine, Shanghai, China; ^3^ Breast Cancer Center, Shanghai East Hospital, Nanjing Medical University, Shanghai, China; ^4^ Breast Cancer Center, Shanghai East Hospital, Tongji University School of Medicine, Shanghai, China

**Keywords:** cervical cancer, recurrence, immune subtypes, immune microenvironment, therapeutic response, PANoptosis

## Abstract

**Objective:** Cervical cancer poses a remarkable health burden to females globally. Despite major advances in early detection and treatment modalities, some patients still relapse. The present study proposed a novel immune molecular classification that reflected distinct recurrent risk and therapeutic responses in cervical cancer.

**Methods:** We retrospectively collected two cervical cancer cohorts: TCGA and GSE44001. Consensus clustering approach was conducted based on expression profiling of recurrence- and immune-related genes. The abundance of immune cells was inferred *via* five algorithms. Immune functions and signatures were quantified through ssGSEA. Genetic mutations were analyzed by maftools package. Immunotherapeutic response was inferred *via* tumor mutation burden (TMB), Tumor Immune Dysfunction and Exclusion (TIDE), and Submap methods. Finally, we developed a LASSO model for recurrence prediction.

**Results:** Cervical cancer samples were categorized into two immune subtypes (IC1, and IC2). IC2 exhibited better disease free survival (DFS), increased immune cell infiltration within the immune microenvironment, higher expression of immune checkpoints, higher activity of immune-relevant pathways (APC co-inhibition and co-stimulation, inflammation-promoting, MHC class I, IFN response, leukocyte and stromal fractions, macrophage regulation, and TCR Shannon), and higher frequencies of genetic mutations. This molecular classification exhibited a remarkable difference with existing immune subtypes, with diverse PANoptosis (pyroptosis, apoptosis and necroptosis) features. Patients in IC2 were more likely to respond to immunotherapy and targeted, and chemotherapeutic agents. The immune subtype-relevant signature was quantified to predict patients’ recurrence risk.

**Conclusion:** Altogether, we developed an immune molecular classification, which can be utilized in clinical practice to aid decision-making on recurrence management.

## Introduction

Cervical cancer poses a significant health burden to females worldwide (Castle et al., 2021). Although this malignancy is preventable and treatable, it remains the fourth most diagnosed cancer as well as the fourth major cause of cancer-related deaths in females (Sung et al., 2021). It is estimated that around 600,000 females are diagnosed with cervical cancer, and over 300,000 females die from this malignancy globally each year (Sung et al., 2021). Most cervical cancer cases correlate to infection with high-risk HPV. Primary treatment of cervical cancer that typically includes surgery, chemoradiation, or their combination has a cure rate of about 95% for early-stage disease as well as 40%–60% for locally advanced disease (Miccò et al., 2022). Recurrent cervical cancer is defined as local tumor regrowth, lymph node or distant metastasis following the primary tumor has regressed for at least 6 months (Miccò et al., 2022). Management of recurrent cervical cancer depends upon treatment history, location, and degree of recurrence. Up to 26% of patients with early-stage cervical cancer relapse following initial surgery (Cibula et al., 2022). Furthermore, many patients with treatment history experience recurrence without symptoms.

Accumulated evidence demonstrates that the complex cellular compositions within the immune microenvironment result in intratumoral heterogeneity (Qiu et al., 2020). Immune-related genes (IRGs) correlate to the immune microenvironment, clinical outcomes as well as treatment response in cervical cancer (Xu et al., 2021). PANoptosis is an inflammatory programmed cell death, which can be activated by components that are simultaneously involved in pyroptosis, apoptosis and/or necroptosis (Karki et al., 2020). If one or more programmed cell death pathways are hindered in tumor cells, PANoptosis is beneficial for tumor suppression *via* inducing mechanisms by which the host activates alternative cell death defense mechanisms (Lee et al., 2021). Currently, the use of immunotherapy to treat cervical cancer is being actively researched, though several immunotherapy drugs (pembrolizumab, etc.), have gained the approval of the FDA (Colombo et al., 2021). Because immunotherapies have established a new standard of care in cervical cancer treatment, novel biomarkers to recognize ideal patient populations for these therapies are of importance. In the present study, in accordance with the expression profiling of recurrence-related IRGs, we proposed a novel immune molecular classification, and classified cervical cancer patients into two immune subtypes with distinct recurrence risk, immune microenvironment as well as immuno-, targeted- and chemotherapeutic responses, thus aiding clinical therapeutic decision-making.

## Materials and methods

### Data collection and processing

RNA sequencing, somatic mutation data (MAF format) and clinical follow-up information of cervical cancer patients were downloaded from the Cancer Genome Atlas (TCGA; https://portal.gdc.cancer.gov/). After thoroughly querying the Gene Expression Omnibus (GEO) database (https://www.ncbi.nlm.nih.gov/gds/), an eligible cervical cancer dataset (GSE44001) with disease recurrence information and genetic profiling was enrolled (Lee et al., 2013). All data were downloaded on 15 April 2022. After removal of samples without recurrence time and status (i.e. disease free survival (DFS)), we included 174 samples in the TCGA dataset and 300 samples in the GSE44001 dataset for subsequent analysis. The demographics and follow-up data were listed in Supplementary Table S1. A total of 782 IRGs were collected from previously published literature, as listed in Supplementary Table S2 (Charoentong et al., 2017). In addition, we collected the gene sets of PANoptosis (pyroptosis, apoptosis and necroptosis) were acquired from previously published literature (Supplementary Table S3) (Pan et al., 2022).

### Unsupervised consensus clustering analysis

Univariate cox regression analysis of IRGs was implemented based on recurrence time and status both in the TCGA and GSE44001 datasets. In accordance with *p* < 0.05, recurrence-related IRGs were obtained, with hazard ratio (HR)>1 as a risk factor and HR < 1 as a protective factor. Then, protective and risk factors of the two datasets were separately intersected for subsequent consensus clustering analysis. Unsupervised consensus clustering was implemented for constructing an immune molecular classification using ConsensusClusterPlus package (version 1.52.0) based on recurrence-related IRGs (Wilkerson and Hayes, 2010). The clustering procedure, with 100 iterations, was carried out by subsampling 80% of the data in each iteration. The optimal number of clusters was identified based on consensus cumulative distribution function (CDF), relative change in area under CDF curve, and consensus heatmap.

### Differential expression and functional enrichment analyses

By limma package (Ritchie et al., 2015), differential expressed genes (DEGs) between subtypes were selected according to |fold change (FC)|>1.5 and false discovery rate (FDR)<0.05. Gene Ontology (GO) functional annotation and Kyoto Encyclopedia of Genes and Genomes (KEGG) pathway enrichment analyses of up-regulated and down-regulated genes were carried out using clusterProfiler package (Yu et al., 2012), respectively, with FDR<0.05 as the threshold value. The GSEA function of clusterProfiler package was also employed to conduct functional enrichment analysis between subtypes. The “c2.cp.kegg.v7.4.symbols.gmt” and “h.all.v7.4.symbols.gmt” gene sets in the Molecular Signatures database (http://www.gsea-msigdb.org/) (Liberzon et al., 2015) were utilized as reference sets.

### Immune cell infiltrations

EPIC (Racle et al., 2017), Estimation of Stromal and Immune cells in Malignant Tumor tissues using Expression data (ESTIMATE) (Yoshihara et al., 2013), MCP-counter (Becht et al., 2016), single-sample gene set enrichment analysis (ssGSEA) (Hänzelmann et al., 2013), and Tumor Immune Estimation Resource (TIMER) (Li et al., 2017) algorithms were implemented for inferring the relative proportions of infiltrating immune cells. The activity of known immune functions or signatures was estimated with ssGSEA approach.

### Mutational analysis

The mutation frequencies of genes were calculated and visualized utilizing maftools package (version 2.4.05) (Mayakonda et al., 2018). Tumor mutation burden (TMB), a promising biomarker for immunotherapeutic response, was computed as the total number of nonsynonymous mutations in the coding region per megabase (Wang et al., 2021).

### Prediction of immunotherapeutic response

Tumor Immune Dysfunction and Exclusion (TIDE) was computed in accordance with two main mechanisms of tumor immune escape: inducing T cell dysfunction in tumor tissue with increased cytotoxic T lymphocyte (CTL) infiltration as well as preventing T cell infiltration within tumor tissue with reduced CTL level (Jiang et al., 2018; Fu et al., 2020). The immunotherapeutic response was predicted with TIDE algorithm on the basis of gene expression profiling. Submap approach was also utilized to infer the responses (complete response (CR), partial response (PR), stable disease (SD), and progressive disease (PD)) to immune checkpoint blockade of PD-1 and CTLA4 from the IMvigor210 cohort (Yang et al., 2020). The demographics and follow-up information of the IMvigor210 cohort was shown in Supplementary Table S4 **.** FDR < 0.05 was regarded as the threshold for a significant response or nonresponse to anti-PD1 or anti-CTLA4 treatment.

### Evaluation of sensitivity to targeted and chemotherapeutic agents

Half maximal inhibitory concentration (IC50) values of AKT inhibitor VIII, Cisplatin, Erlotinib, Lapatinib, Paclitaxel, and Temozolomide were estimated to reflect therapeutic response with pRRophetic package (Geeleher et al., 2014) on the basis of the Genomics of Drug Sensitivity in Cancer database (www.cancerRxgene.org) (Yang et al., 2013), and the prediction accuracy was assessed with 10-fold cross-validation.

### Establishment of an immune subtype-relevant signature

Cervical cancer patients in the GSE44001 dataset were randomly classified into the training and testing sets at a ratio of 1:1. Firstly, in the training set, univariate cox regression analysis of DEGs was conducted by survival package (version 3.1-12), with *p* < 0.05 as recurrence-related DEGs. Afterwards, the least absolute shrinkage and selection operator (LASSO) algorithm was implemented utilizing glmnet package (Engebretsen and Bohlin, 2019). Genes with regression coefficient equal to zero after the shrinkage process were removed. The optimal tuning parameter lambda (λ) was selected when the partial likelihood deviance reached the lowest on the basis of 10-fold cross-validation. The final identified genes were utilized to establish a multivariate cox regression model. The formula of the recurrence model was as follows:

$$RiskScore=\overset{n}{\underset{i=1}{\sum }}coef\left(i\right)*gene(i),$$

where coef(i) represents the coefficient of the i^th^ gene, and gene(i) represents the expression level of the i^th^ gene. RiskScore of each cervical cancer patient was computed. The optimal cutoff was determined *via* surv_cutpoint function of survminer package, and patients were classified into high- and low-risk subgroups. Kaplan-Meier approach was utilized to assess DFS, whereas log-rank test was implemented for assessing recurrence risk. The area under the receiver-operating characteristic curve (AUROC) was used to appraise the predictive capacity of the immune subtype-relevant signature. Above analyses were validated in the GSE44001-testing set, GSE44001-entire set, and independent TCGA dataset. Associations of the signature and clinicopathological parameters with prognosis were estimated through uni- and multivariate cox regression analyses in the TCGA dataset.

### Establishment of a nomogram

A nomogram that integrated the immune subtype-relevant signature and clinicopathological parameters for recurrence prediction was constructed using rms package. The calibration curve was plotted for evaluating the predictive accuracy of the nomogram *via* rms package. Decision curve analysis (DCA) was conducted for determining the clinical application value of the nomogram *via* computing the net benefits at distinct risk thresholds.

### Statistical analysis

Statistical analysis was executed *via* R software (version 4.1.0; https://www.r-project.org/). To compare continuous variables between groups, Student’s t-test was implemented, whereas Wilcoxon test was applied to compare non-normally distributed variables. Through chi-squared test, categorical variables between groups were compared. The two-sided *p* ≤ 0.05 was regarded as statistical significance.

## Results

### Construction of immune subtypes for cervical cancer with different recurrence outcomes based on recurrence-related immune-related genes

**FIGURE 1:**
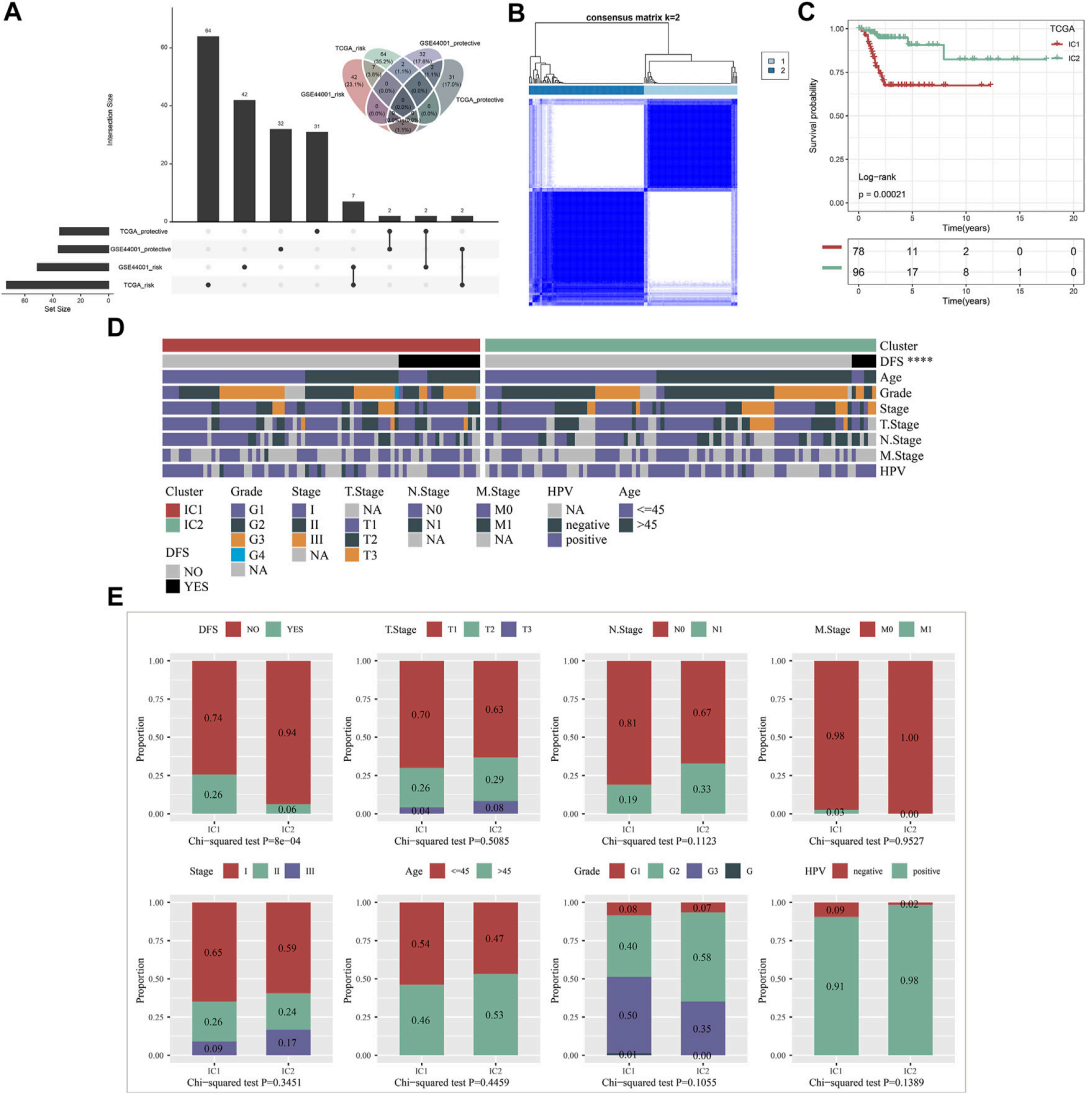
Construction of immune subtypes for cervical cancer with different recurrence outcomes based on recurrence-related IRGs in the TCGA dataset. **(A)** Univariate cox regression analysis of protective factors and risk factors for cervical cancer recurrence among IRGs in the TCGA and GSE44001 datasets. **(B)** Consensus matrix heatmap of cervical cancer samples based on the expression profiling of recurrence-related IRGs when k = 2. **(C)** Kaplan-Meier curves of DFS between IC1 and IC2 in the TCGA dataset. **(D)** Landscape of clinicopathological parameters in two immune subtypes (****p < 0.0001). **(E)** Comparison of DFS, T, N, M, histological stage, age, grade, and HPV between immune subtypes.

Both in the TCGA and GSE44001 datasets, we determined 7 protective factors and 2 risk factors for cervical cancer recurrence among IRGs by implementing univariate cox regression analysis (Figure 1A), which were included for consensus clustering analysis. Consensus matrix heatmap, CDF and relative change in area under CDF curve showed that cervical cancer samples in the TCGA dataset were clearly classified as two immune subtypes (namely IC1 and IC2) (Figure 1B; Supplementary Figures S1A,B). This classification was confirmed in the GSE44001 dataset (Supplementary Figure S2A), indicating that the clustering of samples was stable and reliable. There was a remarkably difference in DFS outcome between immune subtypes both in the TCGA (Figure 1C) and GSE44001 (Supplementary Figure S2B) datasets, with a more favorable DFS outcome for IC2. Clinicopathological features were also compared between immune subtypes. The proportion of non-recurrent patients was remarkably higher in IC2 than that of IC1 both in the TCGA (Figures 1D,E) and GSE44001 (Supplementary Figures S2C–E) datasets.

### Immune subtypes-relevant genes and their biological significance

**FIGURE 2:**
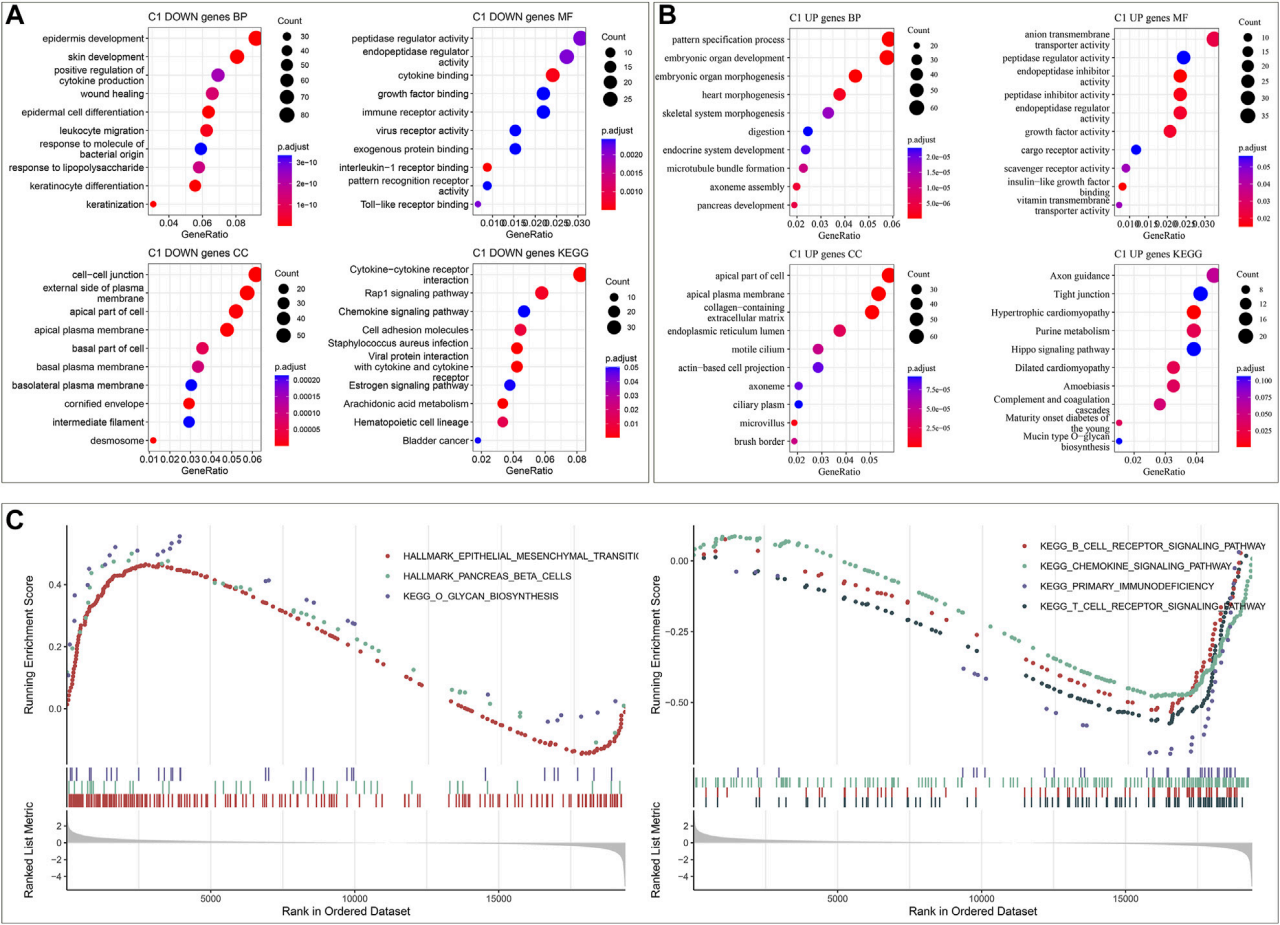
Immune subtypes-relevant genes and their biological significance in the TCGA dataset. **(A)** The main biological process (BP), molecular function (MF), cellular component (CC) and KEGG enrichment results of genes with down-regulation in IC1. **(B)** The main BP, MF, CC and KEGG enrichment results of genes with up-regulation in IC1. **(C)** GSEA for the main Hallmark and KEGG pathways activated in IC1 or IC2.

To unveil the molecular mechanisms underlying immune subtypes, DEGs between IC1 and IC2 were selected with |FC|>1.5 and FDR<0.05. In the TCGA dataset, 1223 DEGs with up-regulation and 976 with down-regulation were determined in IC1 compared with IC2 (Supplementary Table S5). Moreover, 138 DEGs with up-regulation and 539 with down-regulation were investigated in IC1 compared with IC2 in the GSE44001 dataset (Supplementary Table S6). DEGs with down-regulation were remarkably linked with immune-relevant pathways (cytokine, chemokine and their receptor signaling, etc.; Figure 2A; Supplementary Figure S3A). Moreover, DEGs with up-regulation were mainly correlated to cervical cancer-relevant pathways (Hippo signaling pathway, etc.; Figure 2B; Supplementary Figure S3B). GSEA was also employed for pathways activated in IC1 and IC2. Both in the TCGA and GSE44001 datasets, epithelial mesenchymal transition, pancreas beta cells and O-glycan biosynthesis were remarkably activated in IC1 (Figure 2C; Supplementary Figure S3C). Moreover, B cell receptor, chemokine, and T cell receptor signaling pathways and primary immunodeficiency displayed remarkable activation in IC2.

### Immune subtypes with distinct immune microenvironment and immune checkpoints

**FIGURE 3:**
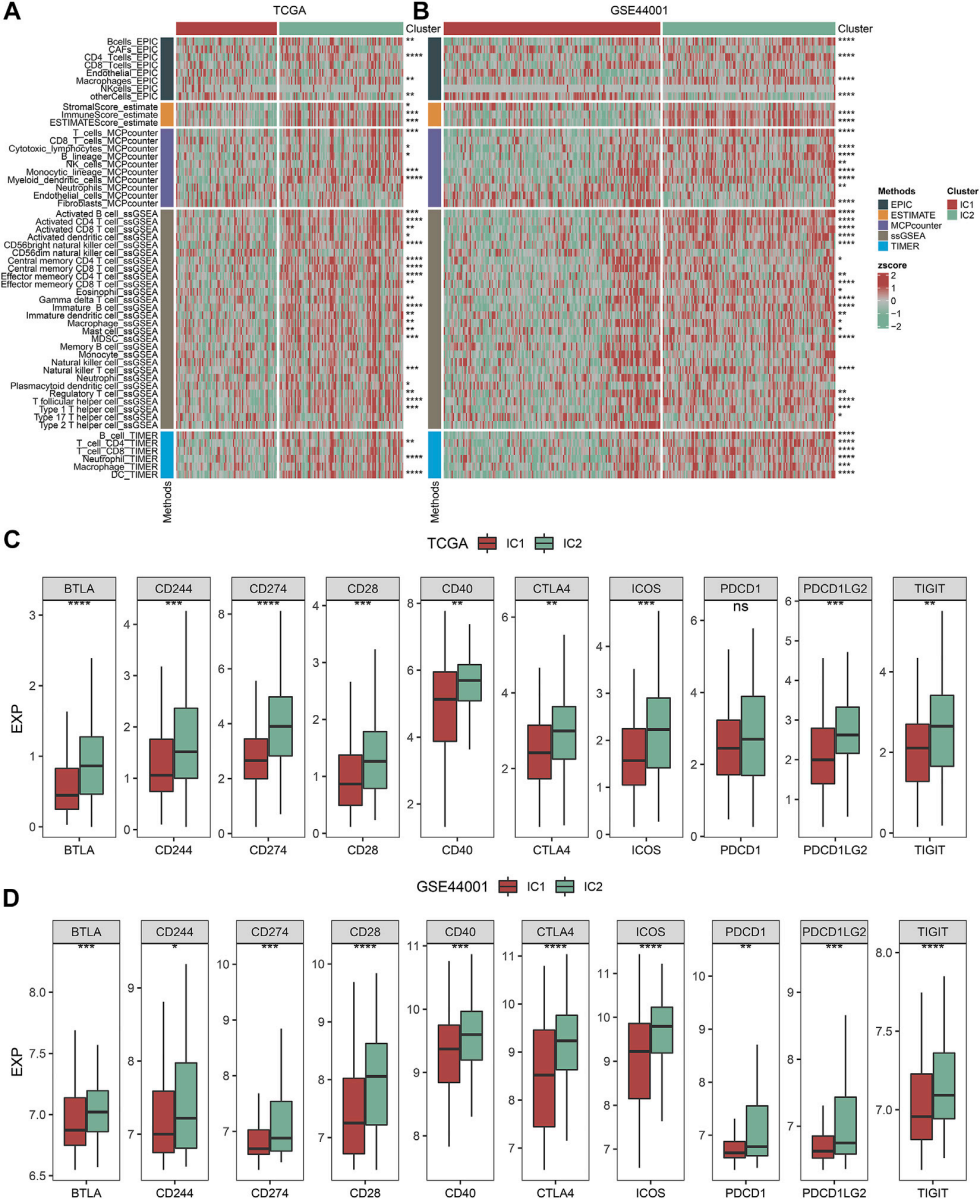
Immune subtypes with distinct immune microenvironment and immune checkpoints. **(A,B)** Heatmaps of the abundance of immune cells inferred by several approaches in IC1 and IC2 immune subtypes in the TCGA and GSE44001 datasets. **(C,D)** Expression of known immune checkpoints in two immune subtypes in the TCGA and GSE44001 datasets (ns: no significance; *p < 0.05; **p < 0.01; ***p < 0.001; ****p < 0.0001).

Five approaches (MCP-counter, ESTIMATE, ssGSEA, EPIC, and TIMER) were applied for inferring the abundance of immune cells across cervical cancer from the TCGA and GSE44001 datasets. The consistent results from distinct approaches showed that IC2 exhibited the higher abundance of immune cells in comparison to IC1 both in two datasets (Figures 3A,B). Moreover, we acquired known immune checkpoints from previously published literature (Danilova et al., 2019; Li et al., 2019; Zheng et al., 2020). Both in the TCGA and GSE44001 datasets, BTLA, CD244, CD274, CD28, CD40, CTLA4, ICOS, PDCD1, PDCD1LG2, and TIGIT displayed higher expression in IC2 in comparison to IC1 (Figures 3C,D).

### Immune subtypes with distinct immune functions

**FIGURE 4:**
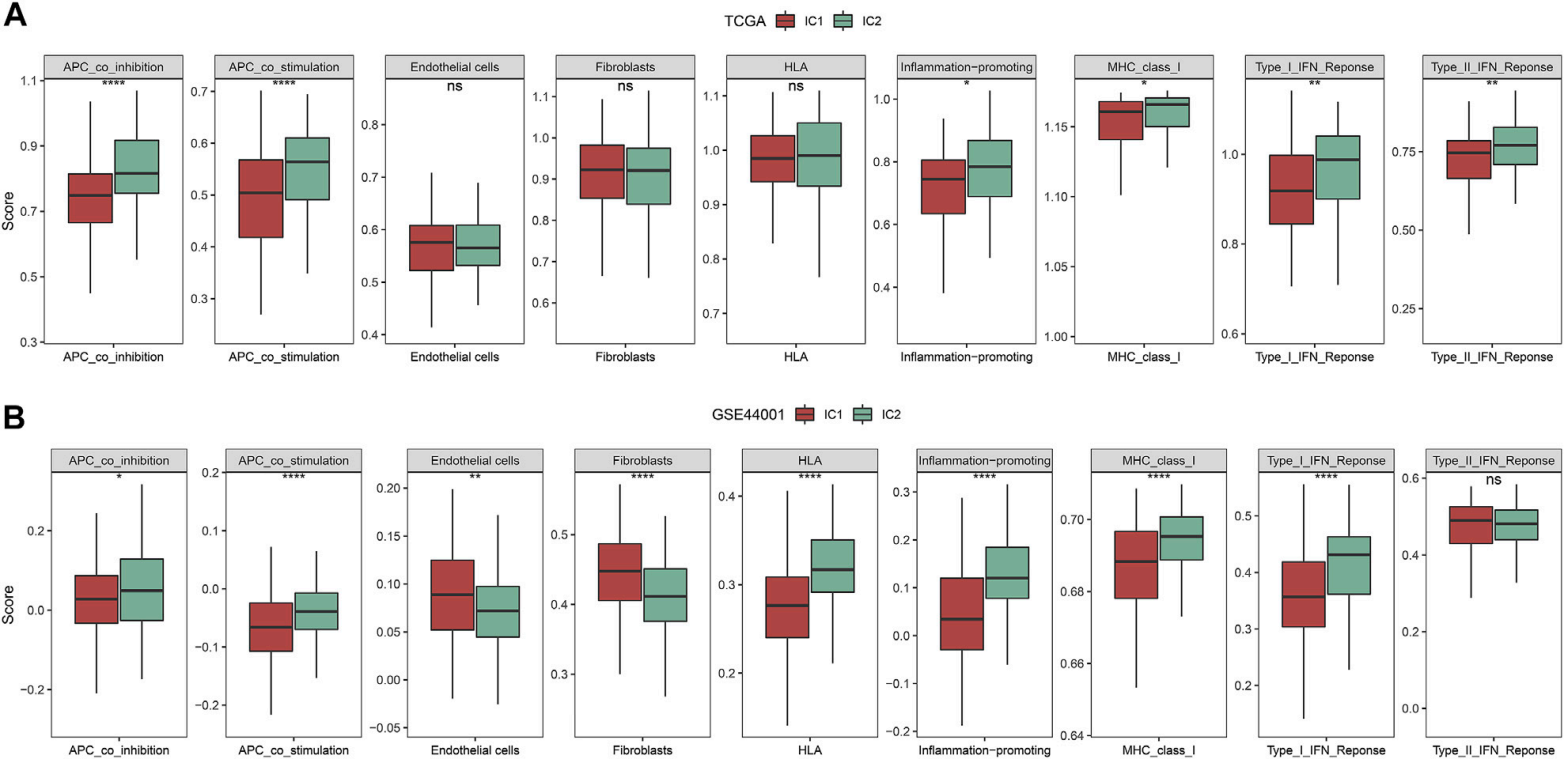
Immune subtypes with distinct immune-relevant pathways. **(A,B)** The activity of immune-relevant pathways in IC1 and IC2 immune subtypes in the TCGA and GSE44001 datasets (ns, no significance; *p < 0.05; **p < 0.01; ****p < 0.0001).

By applying ssGSEA approach, we inferred the activity of immune-relevant pathways across cervical cancer. In comparison to IC1, we observed that APC co-inhibition and co-stimulation, inflammation-promoting, MHC class I, type I and II IFN responses exhibited increased activity in IC2 both in the TCGA and GSE44001 datasets (Figures 4A,B).

### Associations of our molecular classification with existing immune subtypes

**FIGURE 5:**
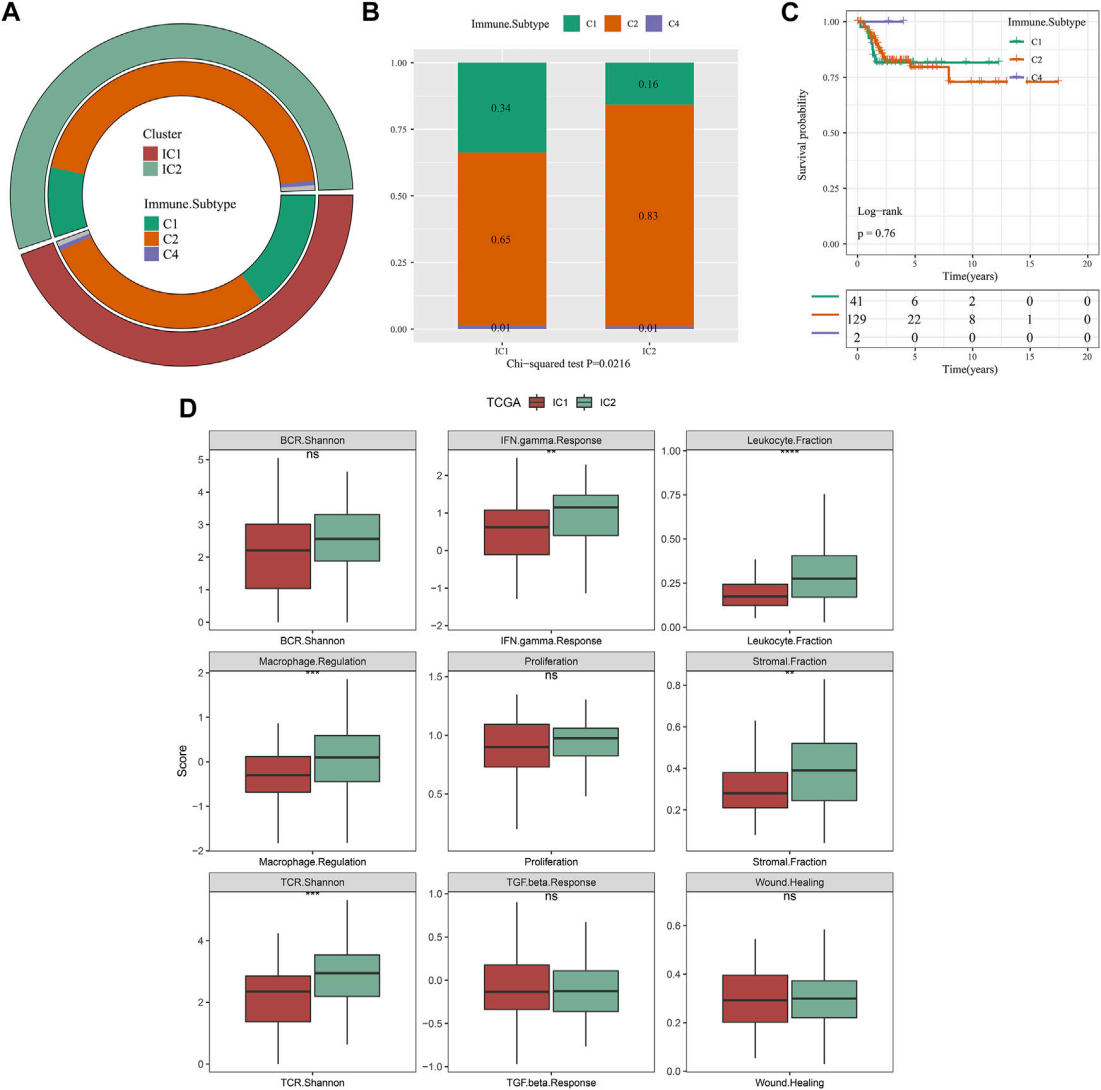
Associations of our molecular classification with existing immune subtypes in the TCGA dataset. **(A)** The distribution of existing immune subtypes (C1, C2 and C4) across our molecular classification. **(B)** Comparison of proportion of existing immune subtypes between IC1 and IC2 immune subtypes. **(C)** Kaplan-Meier curves of DFS among existing immune subtypes (C1, C2 and C4). **(D)** The activity of immune signatures between IC1 and IC2 immune subtypes (ns, no significance; **p < 0.01; ***p < 0.001; ****p < 0.0001).

As depicted in Figure 5A, our molecular classification exhibited a remarkable difference with existing immune subtypes (Ceccarelli et al., 2016). Compared with IC1, higher proportion of C2 and lower proportion of C1 were observed in IC2 (Figure 5B). However, there was no remarkable difference in DFS among existing immune subtypes (C1, C2, and C4) (Figure 5C). Thus, immune subtypes we proposed was a novel molecular classification of cervical cancer different from existing immune subtypes. The activity of immune signatures was compared between IC1 and IC2. Compared with IC1, IFN gamma response, leukocyte fraction, macrophage regulation, stromal fraction, and TCR Shannon exhibited higher activity in IC2 (Figure 5D).

### Immune subtypes with distinct genetic mutations

**FIGURE 6:**
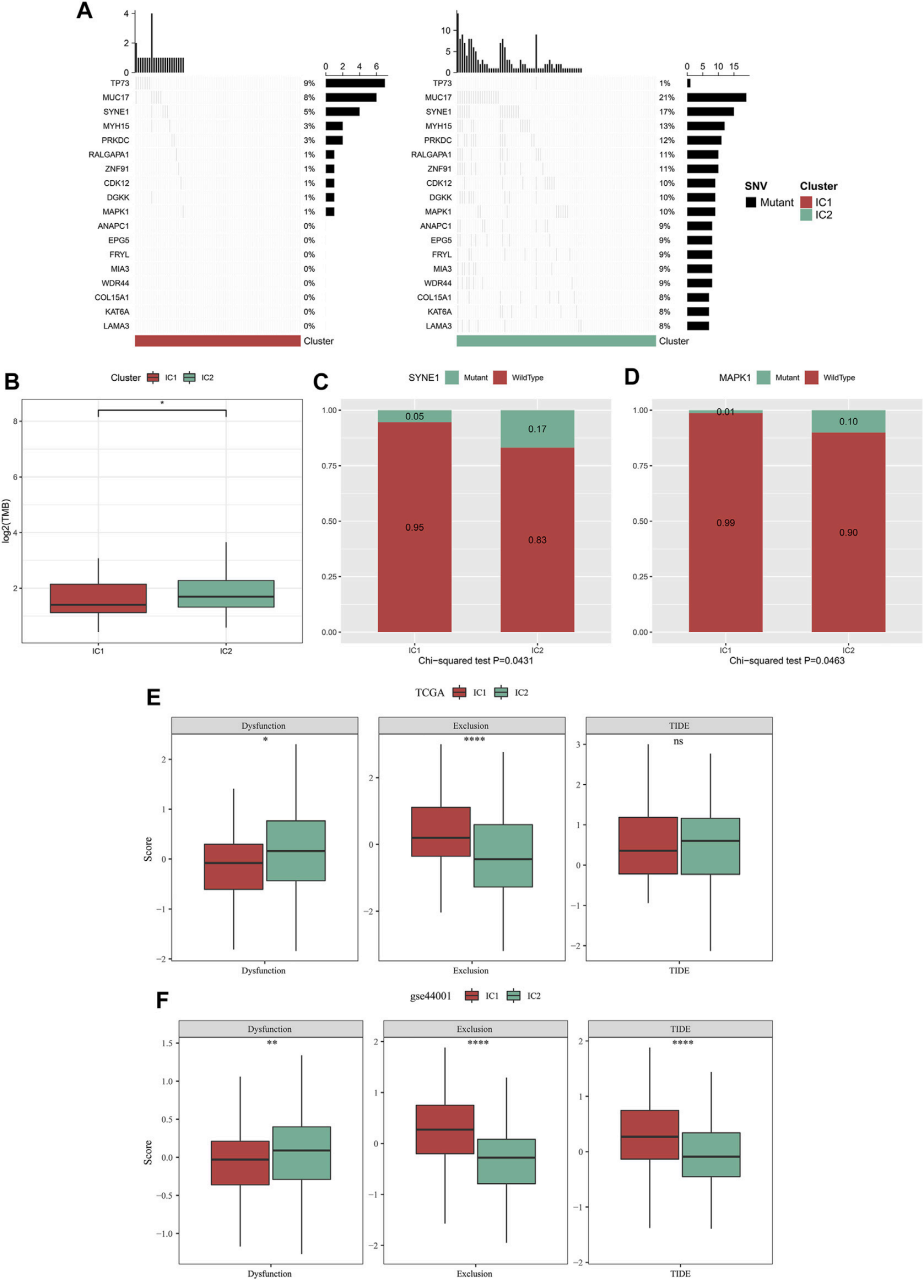
Immune subtypes with distinct genetic mutations and immunotherapeutic responses. **(A)** Waterfall diagrams of the mutational frequencies of genes with remarkable differences between IC1 and IC2 immune subtypes in the TCGA dataset. **(B)** Comparison of TMB between two immune subtypes in the TCGA dataset. **(C,D)** Comparison of mutation frequencies of SYNE1 and MAPK1 between immune subtypes in the TCGA dataset. **(E,F)** Comparison of dysfunction, exclusion, and TIDE score between immune subtypes in the TCGA and GSE44001 datasets (ns, no significance; *p < 0.05; **p < 0.01; ****p < 0.0001).

Through maftools approach, we analyzed and visualized genetic mutations of cervical cancer. The mutation frequencies of the top 500 mutated genes were compared between IC1 and IC2 immune subtypes. With *p* < 0.05, mutations in 18 genes differed significantly between immune clustered (Figure 6A). Compared with IC1, MUC17, SYNE1, MYH15, PRKDC, RALGAPA1, ZNF91, CDK12, DGKK, MAPK1, ANAPC1, EPG5, FRYL, MIA3, WDR44, COL15A1, KAT6A, and LAMA3 displayed higher mutation frequencies in IC2. However, lower mutation frequency of TP73 was observed in IC2. We also computed TMB of each cervical cancer, and analyzed the difference in TMB between immune subtypes. In Figure 6B, compared with IC1, higher TMB score was observed in IC2. Especially, we compared mutation frequencies of SYNE1 and MAPK1 between immune subtypes. Higher mutation frequencies of SYNE1 and MAPK1 were found in IC2 compared with IC1 (Figures 6C,D). Altogether, our data demonstrated that IC2 exhibited higher genetic mutations than IC1.

### Immune subtypes with distinct immunotherapeutic responses

**FIGURE 7:**
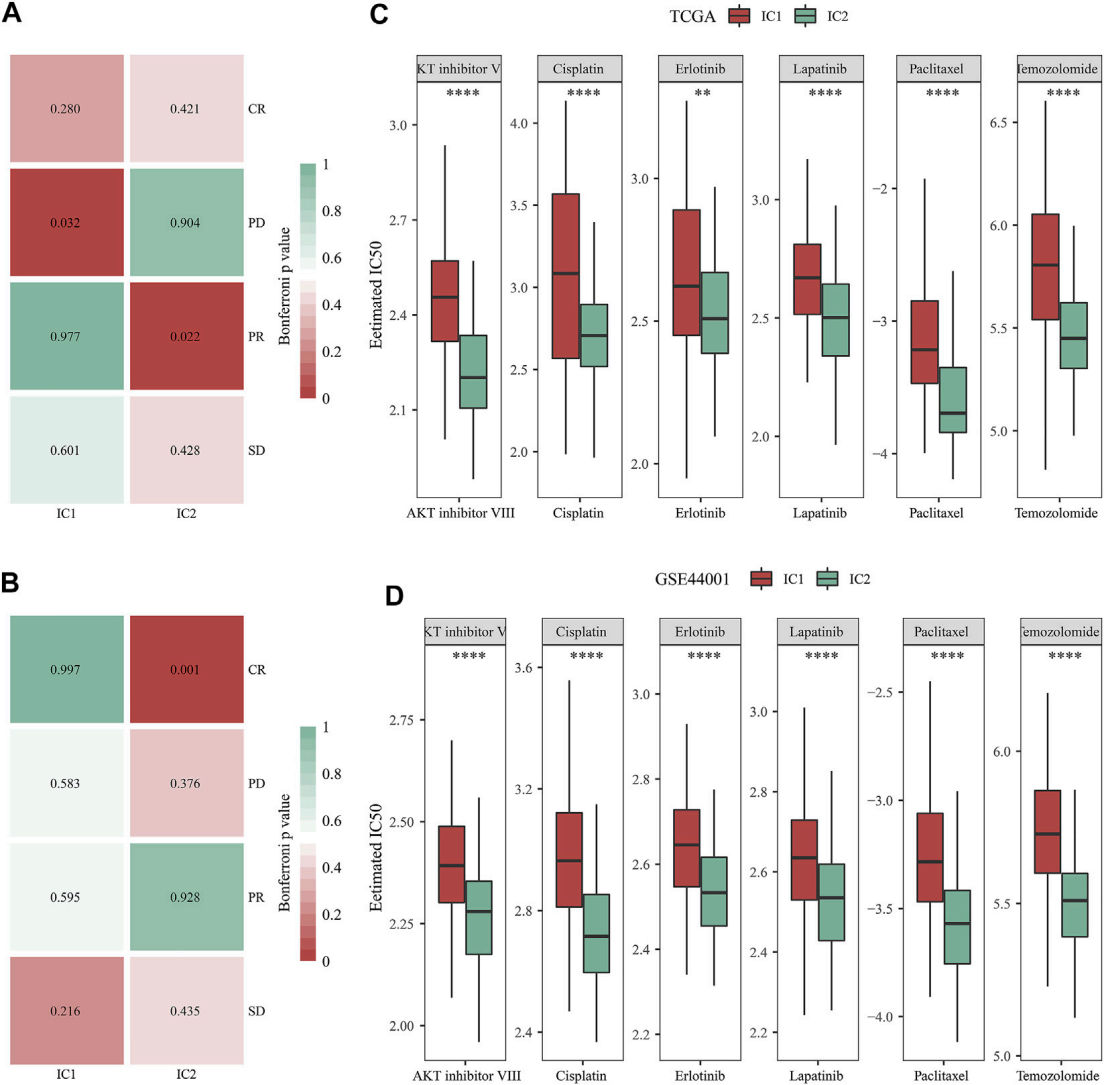
Immune subtypes with distinct immuno-, targeted, and chemotherapeutic responses. **(A,B)** Submap for comparing the expression profiling of two immune subtypes with that of IMvigor210 cohort (including four subsets with different responses to anti-PD-L1 immunotherapy in the TCGA and GSE44001 datasets. **(C,D)** Comparison of the IC50 values of targeted, and chemotherapeutic agents (AKT inhibitor VIII, Cisplatin, Erlotinib, Lapatinib, Paclitaxel, and Temozolomide) between two immune subtypes in the TCGA and GSE44001 datasets (**p < 0.01; ****p < 0.0001).

TIDE is a reliable biomarker to predict response to immunotherapy (Jiang et al., 2018; Fu et al., 2020). The lower TIDE score, the greater the likelihood of immunotherapeutic response. Here, we computed TIDE in cervical cancer from the TCGA and GSE44001 datasets. Higher dysfunction, and lower exclusion score were observed in IC2 than IC1 (Figures 6E,F) both in two datasets. There was no remarkable difference in TIDE score between immune subtypes in the TCGA dataset. Differently, IC2 exhibited lower TIDE score in comparison to IC1. Altogether, our data demonstrated that patients in IC2 were more likely to respond to immunotherapy. Furthermore, we employed Submap approach to compare the expression profiling of two immune subtypes with that of the IMvigor210 anti-PD-L1 immunotherapy dataset. Both in the TCGA and GSE44001 datasets, the expression profiling of IC2 was similar to that of patients who responded to anti-PD-L1 immunotherapy (Figures 7A,B). Thus, patients in IC2 were more suitable for immunotherapy, which were similar to TIDE prediction.

### Immune subtypes with distinct targeted, and chemotherapeutic responses

Then, we analyzed the difference in targeted, and chemotherapeutic responses between IC1 and IC2 immune subtypes. Both in the TCGA and GSE44001 datasets, lower IC50 values of AKT inhibitor VIII, Cisplatin, Erlotinib, Lapatinib, Paclitaxel, and Temozolomide were observed in IC2 compared with IC1 (Figures 7C,D). This indicated that patients in IC2 were more likely to respond to above targeted, and chemotherapeutic agents.

### Construction of an immune subtype-relevant recurrence model for cervical cancer

**FIGURE 8:**
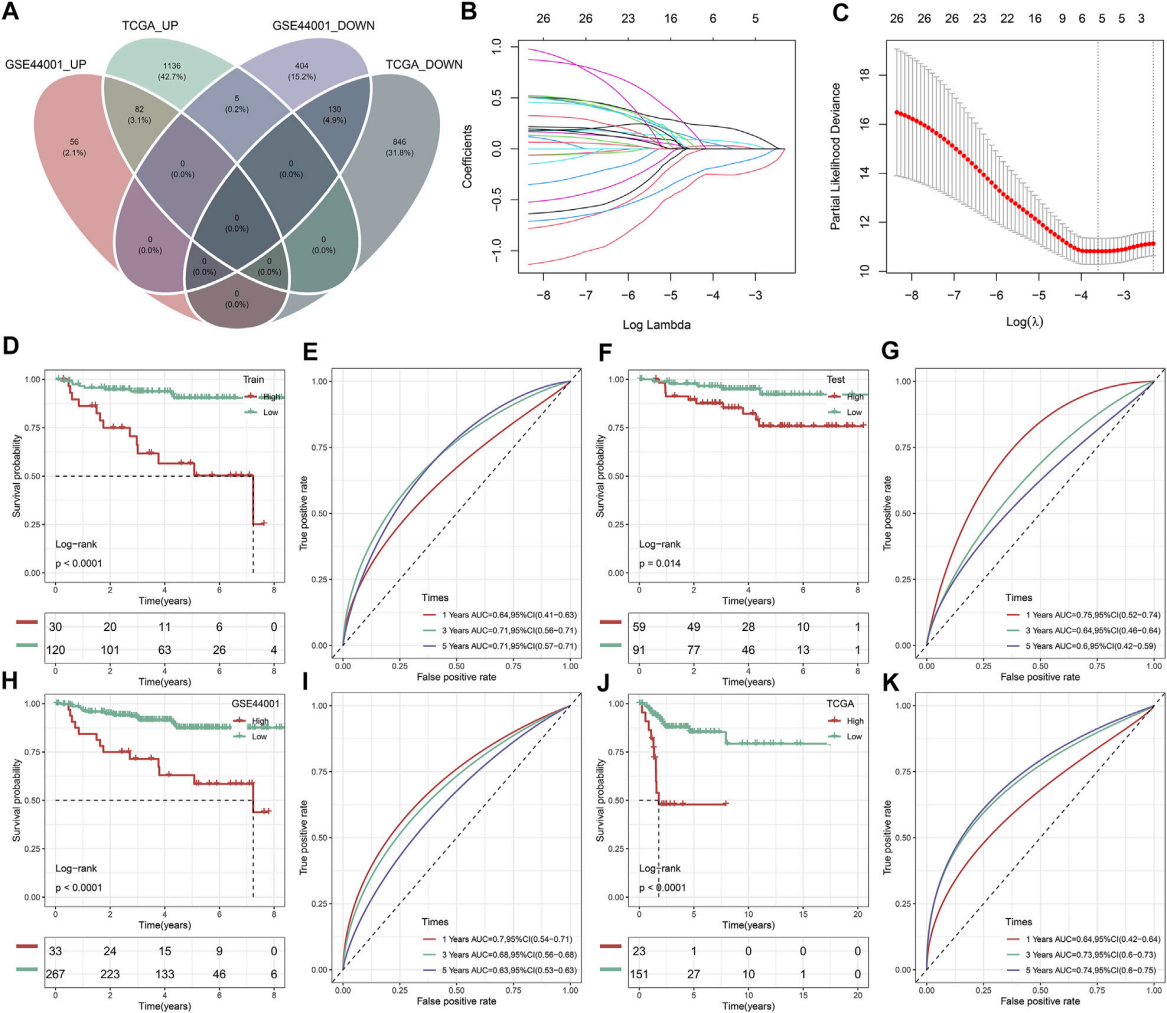
Construction and verification of an immune subtype-relevant recurrence model for cervical cancer. **(A)** Venn diagram of shared DEGs between IC1 and IC2 immune subtypes both in the TCGA and GSE44001 datasets. **(B)** Landscape of regression coefficients of 26 recurrence-related DEGs in the training set. **(C)** Selection of the optimal tuning parameter λ *via* 10-fold cross-validation. Kaplan-Meier curves of DFS and ROC curves at 1-, 3-, and 5-year DFS in the **(D,E)** GSE44001 training set, **(F,G)** GSE44001 testing set, **(H,I)** GSE44001 dataset and **(J,K)** TCGA dataset based on the risk score.

A total of 212 DEGs between immune subtypes were shared by the TCGA and GSE44001 datasets (Figure 8A). Among them, 26 DEGs were remarkably linked with recurrence of cervical cancer, which were used for subsequent recurrence model establishment (Supplementary Table S7). The GSE44001 dataset was randomly classified into the training and testing sets. In the training set, we applied LASSO algorithm to remove DEGs with regression coefficient equal to zero (Figure 8B). Moreover, based on 10-fold cross-validation, the optimal tuning parameter λ was 0.0271963 when the partial \likelihood deviance reached the lowest (Figure 8C). Finally, TMEM125, TFF1, DECR2, LONRF3, DAPL1, and ANKRD35 were included for establishing a multivariate cox regression model. By combining regression coefficients and expression values of above DEGs, we computed risk score of cervical cancer cases (Table 1). With the optimal cutoff, patients were classified into high- and low-risk subgroups. In Figure 8D, we observed that high-risk cases exhibited worse DFS in comparison to low-risk cases in the training set. AUROC values at 1-, 3- and 5-year DFS were all >0.60, demonstrating the excellent performance of this model in predicting recurrence (Figure 8E). The similar results were observed in the testing set (Figures 8F,G), the GSE44001 (Figures 8H,I) and TCGA (Figures 8J,K) datasets. Thus, the immune subtype-relevant recurrence model exhibited the favorable efficiency in predicting cervical cancer recurrence.

**TABLE 1:** Multivariate cox regression results in the training set.

Gene	coef	HR	HR (lower, 0.95)	HR (upper, 0.95)	P
TMEM125	0.26809	1.3075	0.7677	2.227	0.3237
TFF1	0.02287	1.0231	0.7495	1.397	0.8855
DECR2	0.14912	1.1608	0.6763	1.992	0.5885
LONRF3	0.11064	1.117	0.5829	2.141	0.7388
DAPL1	−0.26837	0.7646	0.5736	1.019	0.0673
ANKRD35	−0.23696	0.789	0.4803	1.296	0.3494

Abbreviations: coef, coefficient; HR, hazard ratio.

### The immune subtype-relevant recurrence model independently predicts cervical cancer recurrence

**FIGURE 9:**
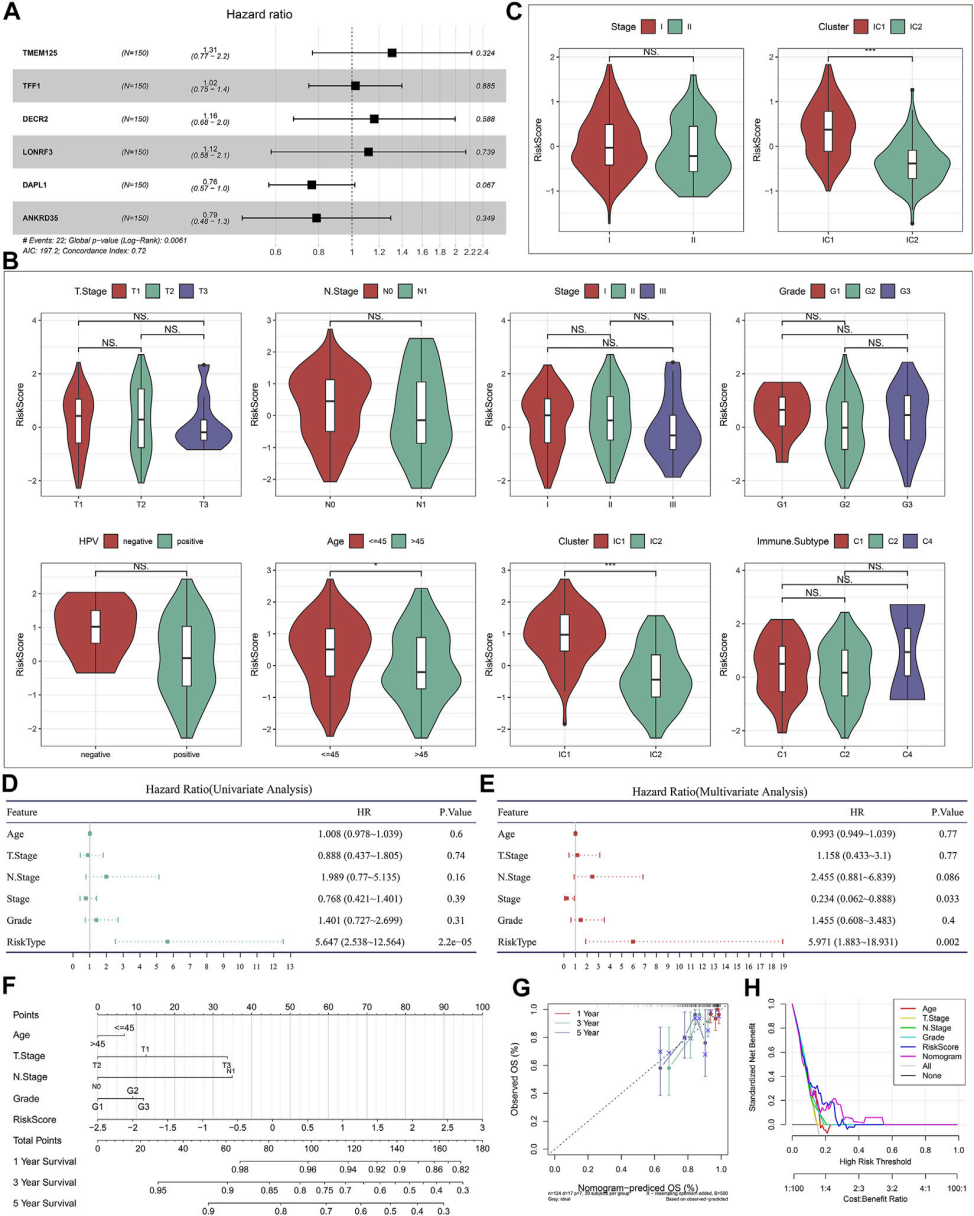
Evaluation of the clinical significance of the immune subtype-relevant recurrence model and establishment of a nomogram for cervical cancer. **(A)** Forest diagram of multivariate cox regression analysis of each variable in the recurrence model in the TCGA dataset. **(B)** Distribution of the risk score across distinct clinicopathological parameters in the TCGA dataset. **(C)** Distribution of the risk score across distinct clinicopathological parameters in the GSE44001 dataset. **(D,E)** Uni- and multivariate cox regression analysis for the associations of the risk score and clinicopathological parameters with DFS in the TCGA dataset. **(F)** Establishment of a nomogram including independent variables in the TCGA dataset. **(G)** Calibration curves for assessing the nomogram-predicted and actual survival outcome. **(H)** Decision curve analysis for evaluation of the net benefit.

Then, we observed that concordance index (C-index) was 0.72, indicating the high prediction accuracy of the recurrence model (Figure 9A). In the TCGA dataset, patients with ≤45 had the relatively higher risk score than those with >45 (Figure 9B). Both in the TCGA and GSE44001 datasets, IC1 exhibited the higher risk score than IC2 (Figures 9B,C). Uni- and multivariate cox regression analysis demonstrated that the model independently predicted cervical cancer recurrence (Figures 9D,E).

### Establishment of a nomogram for cervical cancer recurrence

To facilitate clinical application of the immune subtype-relevant recurrence model, we established a nomogram comprising the risk score and other clinicopathological parameters (Figure 9F). As demonstrated by calibration curves, the nomogram-predicted DFS exhibited the relatively high consistency to actual outcomes (Figure 9G). The net benefits of the nomogram were better than other clinicopathological parameters (Figure 9H), indicating the excellent clinical usefulness.

### PANoptosis features of immune subtypes and immune subtype-relevant recurrence model

**FIGURE 10:**
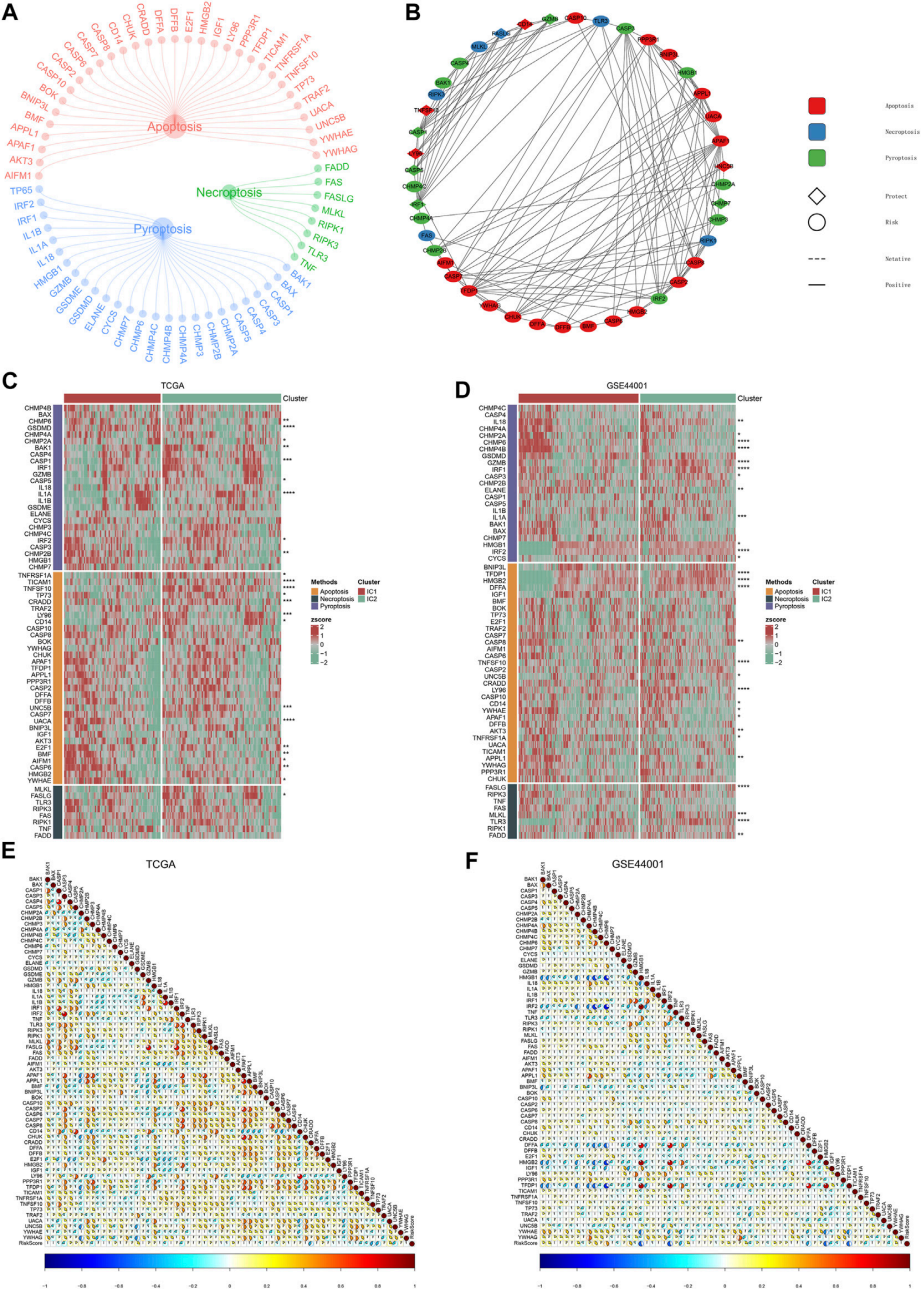
PANoptosis features of immune subtypes and immune subtype-relevant recurrence model. **(A)** The network of PANoptosis (pyroptosis, apoptosis, and necroptosis) genes. **(B)** Prognostic significance of PANoptosis genes and their interactions. **(C,D)** Heatmaps of the expression patterns of PANoptosis genes across two subtypes. **(E,F)** Correlations between immune subtype-relevant recurrence model-derived risk score and PANoptosis genes (*p < 0.05; **p < 0.01; ***p < 0.001; ****p < 0.0001).

Accumulated evidence demonstrates that pyroptosis, apoptosis and necroptosis (PANoptosis) participate in cancer immunity (Pan et al., 2022). Most of PANoptosis genes were significantly linked to cervical cancer prognosis (Figures 10A,B). In addition, there were notable interactions between PANoptosis genes. Both in TCGA and GSE44001 datasets, most pyroptosis, apoptosis and necroptosis genes displayed notable differential expression between immune subtypes (Figures 10C,D). In addition, the immune subtype-relevant recurrence model-derived risk score was significantly correlated to PANoptosis genes in two datasets (Figures 10E,F).

## Discussion

Cervical cancer remains a major gynecological issue globally (Mittal et al., 2022). Despite major advances in early detection and treatment modalities, some patients still relapse. Clinical management of recurrent cervical cancer depends upon treatment history, location as well as degree of recurrence (Zhang et al., 2022). Recurrent cervical cancer usually exhibits challenges for clinicians due to undesirable survival outcomes and limited therapeutic options (Grau et al., 2020). Here, cervical cancer samples were categorized as two immune subtypes with distinct recurrence risk. The novel immune molecular classification was different from existing immune subtypes (Ceccarelli et al., 2016).

Immunoregulators experience immune editing when tumor cells enable to escape immunological surveillance, permit unchecked growth as well as spread (O'Donnell et al., 2019). Also, tumor cells usually apply naturally occurring immunoregulators to escape immune surveillance as well as construct an immunosuppressive microenvironment, whereas lowering anti-tumor activity *via* effector T cells. The results from five algorithms revealed that IC2 exhibited the higher abundance levels of immune cells within the immune microenvironment than IC1. Immune checkpoints can be regulated *via* agonist or antagonist monoclonal antibodies for enhancing T cell activation as well as eliminating suppression of T cell activation, thereby reactivating T cells to attack tumor cells (van der Leun et al., 2020). Recent clinical trials showed that survival outcomes were remarkably longer with cemiplimab, anti-PD-1 inhibitor, compared with single-agent chemotherapy for patients with recurrent cervical cancer following the first-line platinum treatment (Tewari et al., 2022). Moreover, dual PD-1 and CTLA-4 blockage combination displayed durable clinical activity and favorable tolerability as the second-line therapeutic regimen for advanced cervical cancer (O'Malley et al., 2022). However, how to predetermine which patients will respond to immunotherapy remains an issue. Here, we observed that IC2 exhibited higher expression of immune checkpoints (BTLA, CD244, CD274, CD28, CD40, CTLA4, ICOS, PDCD1, PDCD1LG2, and TIGIT) and higher activity of immune-relevant pathways (APC co-inhibition and co-stimulation, inflammation-promoting, MHC class I, IFN response, leukocyte and stromal fractions, macrophage regulation, and TCR Shannon). Evidence indicates that PANoptosis may open an additional avenue for developing promising novel strategies cancer GC immunotherapy. Herein, two immune subtypes exhibited distinct PANoptosis features, and immune subtype-relevant recurrence model-derived risk score correlated to PANoptosis. In accordance with higher TMB, lower TIDE and higher similarity to the expression profiling of patients who well responded to immunotherapy, patients in IC2 were more suitable for immune checkpoint blockade.

Concurrent chemoradiotherapy remains the standard of care for patients with FIGO stage IB 2 or higher (Mittal et al., 2022). Among them, cisplatin is the best-studied and most active single chemotherapeutic drug. Additionally, targeted therapy (anti-angiogenic agent) as well as tyrosine kinase inhibitors have been applied for treating recurrent or metastatic patients. IC2 patients were more likely to respond to targeted, and chemotherapeutic agents (comprising AKT inhibitor VIII, Cisplatin, Erlotinib, Lapatinib, Paclitaxel, and Temozolomide). Currently, CEA, CA125 and SCC remain three major biomarkers of cervical cancer for early screening, treatment monitoring as well as prognostic evaluation (Cao et al., 2022). However, because they exhibit low sensitivity and specificity as expected, novel biomarkers with high reliability, sensitivity and specificity are needed. In the present study, the immune subtype-relevant signature (covering TMEM125, TFF1, DECR2, LONRF3, DAPL1, and ANKRD35) was quantified, which predicted cervical cancer recurrence accurately and independently. Nonetheless, no studies have reported the roles of the genes derived from the signature in cervical cancer. Also, to facilitate clinical practice, we established a nomogram that comprising the immune subtype-relevant signature and known clinicopathological parameters. Despite this, this is a retrospective analysis based on two large cohorts. We will verify above findings in a prospective, and larger cohort in our future research.

## Conclusion

Collectively, our findings proposed a novel immune molecular classification for cervical cancer, which classified cervical cancer patients into two immune subtypes with distinct recurrence risk, immune microenvironment, PANoptosis features as well as immuno-, targeted- and chemotherapeutic responses. Altogether, our findings might aid clinicians to make clinical therapeutic regimens for cervical cancer patients and facilitate personalized precision medicine.

## Data Availability

The datasets presented in this study can be found in online repositories. The names of the repository/repositories and accession number(s) can be found in the article/Supplementary Material.

## Supplementary material

SUPPLEMENTARY FIGURE S1 Identification of the optimal number of clusters. **(A)** Consensus CDF. **(B)** Relative change in area under CDF curve.

SUPPLEMENTARY FIGURE S2 Validation of immune subtypes for cervical cancer with different recurrence outcomes in the GSE44001 dataset. **(A)** Consensus matrix heatmap of cervical cancer samples in accordance with the expression values of recurrence-related IRGs when k = 2. **(B)** Kaplan-Meier curves of DFS between IC1 and IC2. **(C)** Landscape of clinicopathological parameters in two immune subtypes (***p* < 0.01). **(D,E)** Comparison of DFS, and histological stage between immune subtypes.

SUPPLEMENTARY FIGURE S3 Validation of immune subtypes-relevant genes and their biological significance in the GSE44001 dataset. **(A)** The main biological process (BP), molecular function (MF), cellular component (CC) and KEGG enrichment results of genes with down-regulation in IC1. **(B)** The main BP, MF, CC and KEGG enrichment results of genes with up-regulation in IC1. **(C)** GSEA for the main Hallmark and KEGG pathways activated in IC1 or IC2.

SUPPLEMENTARY TABLE S1 The demographics and follow-up data of cervical cancer samples in the TCGA and GSE44001 datasets.

SUPPLEMENTARY TABLE S2 The list of 782 immune-related genes.

SUPPLEMENTARY TABLE S3 The gene sets of PANoptosis (pyroptosis, apoptosis and necroptosis).

SUPPLEMENTARY TABLE S4 The demographics and follow-up information of the IMvigor210 cohort.

SUPPLEMENTARY TABLE S5 The information of DEGs between IC1 and IC2 immune subtypes in the TCGA dataset.

SUPPLEMENTARY TABLE S6 The information of DEGs between IC1 and IC2 immune subtypes in the GSE44001 dataset.

SUPPLEMENTARY TABLE S7 DEGs that were significantly linked with recurrence of cervical cancer.

## Abbreviations

IRGs, immune-related genes; TCGA, the Cancer Genome Atlas; GEO, Gene Expression Omnibus; DFS, disease free survival; HR, hazard ratio; CDF, cumulative distribution function; DEGs, differential expressed genes; FC, fold change; FDR, false discovery rate; GO, Gene Ontology; KEGG, functional annotation and Kyoto Encyclopedia of Genes and Genomes; TMB, tumor mutation burden; TIDE, Tumor Immune Dysfunction and Exclusion; CTL, cytotoxic T lymphocyte; CR, complete response; PR, partial response; SD, stable disease; PD, progressive disease; IC50, half maximal inhibitory concentration; LASSO, least absolute shrinkage and selection operator; AUROC, area under the time-dependent receiver-operating characteristic; DCA, decision curve analysis.
