# Supplementary figures and images for: Definition of immune molecular subtypes with distinct immune microenvironment, recurrence, and PANoptosis features to aid clinical therapeutic decision-making
Source: Front Genet. 2022 Oct 13;13:1007108. doi: 10.3389/fgene.2022.1007108 (PMC9606342; doi:10.3389/fgene.2022.1007108)

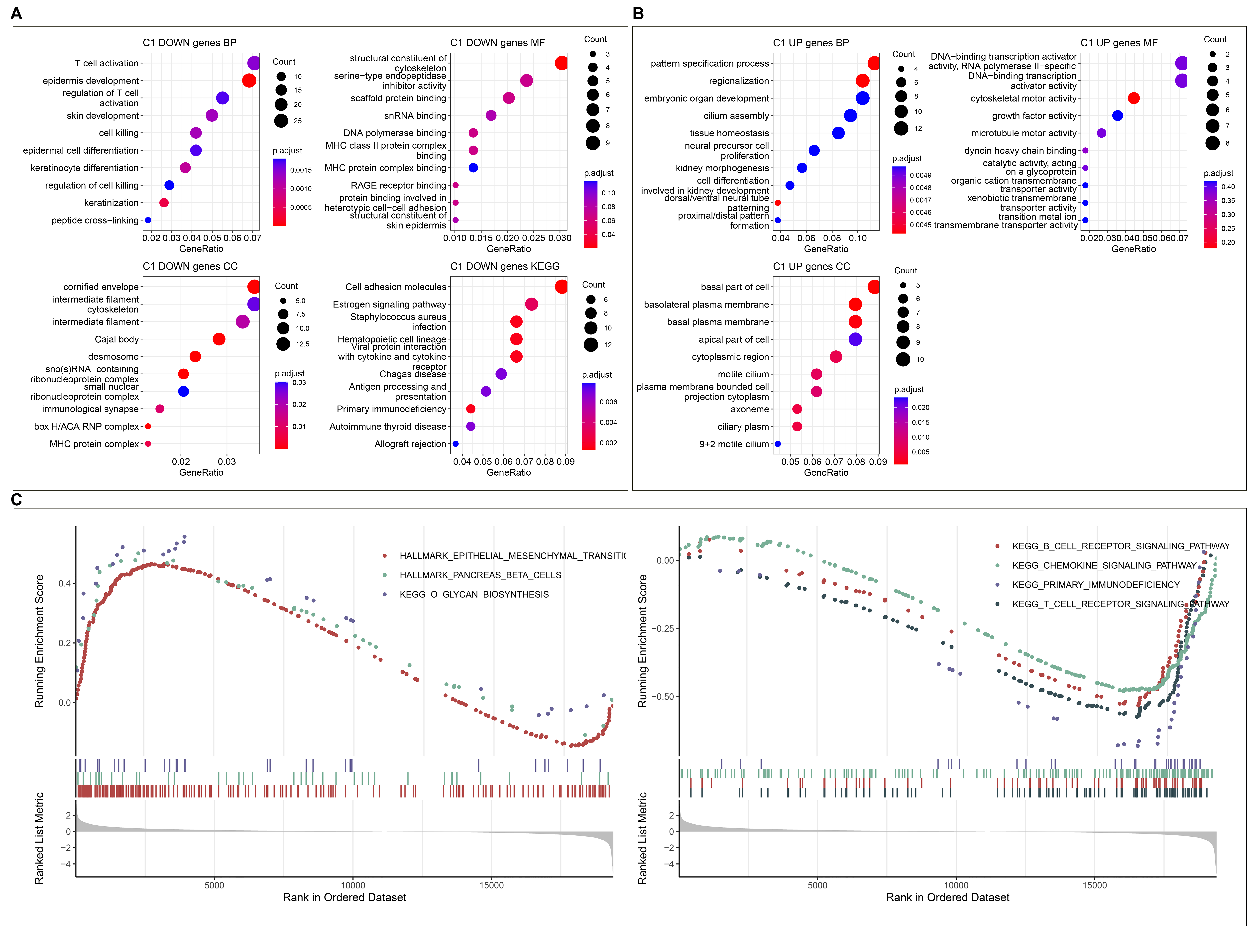

Supplement: Supplementary file 3 [file Image3.TIF]

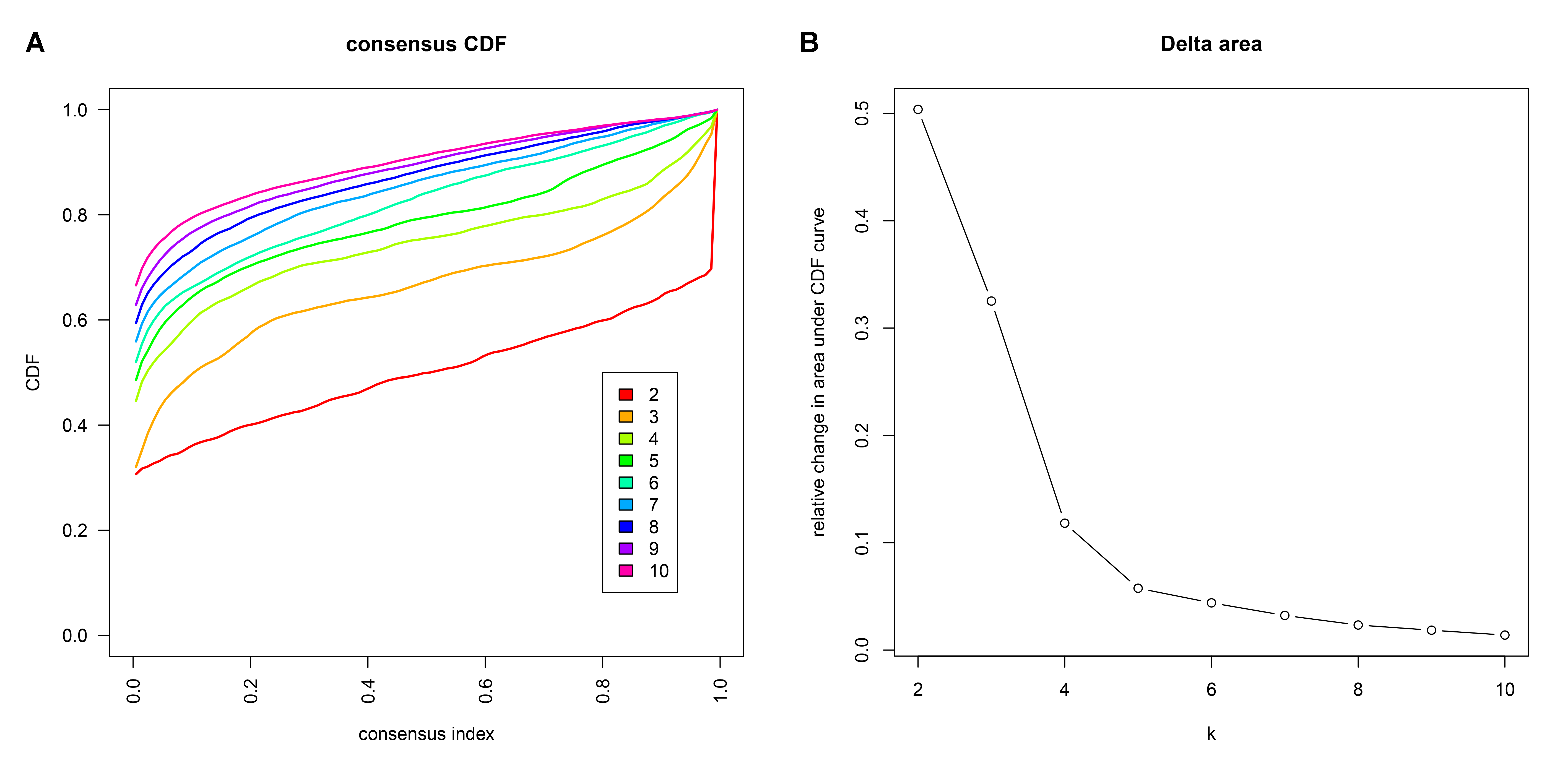

Supplement: Supplementary file 5 [file Image1.TIF]
